# Disease concepts and treatment by tribal healers of an Amazonian forest culture

**DOI:** 10.1186/1746-4269-5-27

**Published:** 2009-10-12

**Authors:** Christopher N Herndon, Melvin Uiterloo, Amasina Uremaru, Mark J Plotkin, Gwendolyn Emanuels-Smith, Jeetendra Jitan

**Affiliations:** 1 Division of Reproductive Endocrinology and Infertility, Department of Obstetrics, Gynecology & Reproductive Sciences, University of California, San Francisco, USA; 2 Amazon Conservation Team Suriname, Nickeriestraat #4, Paramaribo, Suriname; 3 Trio indigenous community of Kwamalasamutu, Sipaliwini District, Suriname; 4 Amazon Conservation Team, 4211 N. Fairfax Drive, Arlington, Virginia, USA; 5 Ministry of Health, Henck Arronstraat 60, Paramaribo, Suriname

## Abstract

**Background:**

The extensive medicinal plant knowledge of Amazonian tribal peoples is widely recognized in the scientific literature and celebrated in popular lore. Despite this broad interest, the ethnomedical systems and knowledge of disease which guide indigenous utilization of botanical diversity for healing remain poorly characterized and understood. No study, to our knowledge, has attempted to directly examine patterns of actual disease recognition and treatment by healers of an Amazonian indigenous culture.

**Methods:**

The establishment of traditional medicine clinics, operated and directed by elder tribal shamans in two remote Trio villages of the Suriname rainforest, presented a unique investigational opportunity. Quantitative analysis of clinic records from both villages permitted examination of diseases treated over a continuous period of four years. Cross-cultural comparative translations were articulated of recorded disease conditions through ethnographic interviews of elder Trio shamans and a comprehensive atlas of indigenous anatomical nomenclature was developed.

**Results:**

20,337 patient visits within the period 2000 to 2004 were analyzed. 75 disease conditions and 127 anatomical terms are presented. Trio concepts of disease and medical practices are broadly examined within the present and historical state of their culture.

**Conclusion:**

The findings of this investigation support the presence of a comprehensive and highly formalized ethnomedical institution within Trio culture with attendant health policy and conservation implications.

## Background

The comparative study of health and medical systems of tribal cultures has contributed broadly to the understanding of disease and development of therapeutics within our own society. The investigation of the *kuru *disease phenomenon among the cannibalistic Fore of New Guinea provided initial insights that led to elucidation of prions, an entirely novel infectious agent [1,2]. Observations of medicinal plant treatments by tribal peoples worldwide have contributed to the development of some of the most important and widely utilized pharmaceutical agents in our medical system [3].

Early travellers to the Amazon were impressed at the number of medicinal plants known to its native peoples [4]. Ethnobotanical inventories derived through survey methodology widely support this perception, with tribal pharmacopoeias often representing dozens, if not hundreds, of botanical species with reputed medicinal value [5-9]. In comparison to studies on ritual and symbolism in shamanism, which are well represented in the literature, few investigations have examined to any extent the understanding of disease and diagnostic processes of Amazonian healers. Important exceptions include Izquierdo and Shepard's body of work examining the ethnomedicine of the Matsigenka culture of the upper Peruvian Amazon and Wilbert's study of Warao concepts and response to epidemic exotic disease in the Venezuelan Orinoco [10-12].

Full consideration of indigenous disease conditions and ethnomedicine, however, may be critical toward understanding the rational selection of therapeutics by tribal healers. Studies among the Tsimane' and Kayapó, for example, demonstrate that tribal healers classify and select medicinal plants according to their principal disease indications [13,14]. Sensory perception of illness may also guide selection of botanical therapeutics by native healers [15,16]. Despite this relevance, published inventories of Amazonian medical ethnobotany rarely present indigenous disease concepts or attempt to develop formalized cross-cultural translations beyond single word approximates, *e.g*., rash or parasites [17].

Outside of Amazonia, Vandebroek et al. broadly examined patterns in the treatment of health conditions by Quechua healers in rural communities of the Bolivian Andes through comparison of the citation frequency of plant-use reports derived through interviews of traditional healers [18]. The few published studies of Amazonian cultures describing patterns of traditional health practice comprise indirect and cross-sectional assessments [13,19,20]. Several investigators have surveyed distribution of self-reported ailments among Tsimane' communities in lowland Bolivia [13,21,22]. Preferences in Tsimane' selection of medicinal plants and Western biomedical treatments for common gastrointestinal afflictions were examined through use of free-listing survey assessments [13].

No investigation, to our knowledge, has attempted to directly examine patterns of actual disease recognition and treatment by traditional healers of an Amazonian forest culture. Our study, utilizing an original methodology, longitudinally investigates disease conditions treated and medicinal plant utilization over a continuous four-year period by elder shamans in two villages of the Trio tribe, a group inhabiting the headwaters of the Suriname-Brazil border frontier region. This paper, the first report from our investigation, focuses on disease conditions identified and treated by Trio healers. Medicinal plant utilization will be subsequently presented and examined in separate publications.

### The Trio

The Trio (syn. Tarëno, Tirio, Tiriyó), characteristic of Carib tribes of the Guiana rainforest, lived principally as shifting cultivators within acephalous groups led by a charismatic elder without strong authoritarian rule [23,24]. Ethnographic evidence indicate, at the time of sustained contact by missionaries in the early 1960s, a remarkably high degree of isolation from non-indigenous exposure, attributable to the geographic in accessibility of their range [25]. Serologic studies of the Trio performed at that time of contact in fact demonstrate lower antibody titers to the influenza virus than had been previously encountered with the exception of populations from certain South Pacific atolls [25].

**Figure 1:**
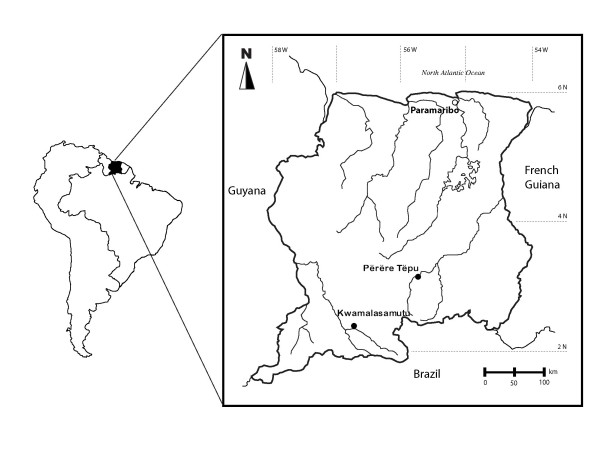
**Location of the study sites**.

The opening up of the region to air travel in 1960s, however, marked the beginning of an on-going period of permanent contact and abrupt cultural and societal change [24]. American and Franciscan missionaries convinced the members of the scattered bands to merge into larger nucleated settlements [26]. At present, the Trio reside principally in three villages (Figure 1), Kwamalasamutu (pop. 1174) and Përëre Tepü (pop. 503) in Suriname and Missão Tiriyo in Brazil (pop. 808). In our paper, the descriptive "Amazonian" is utilized at points in reference to the Trio culture. Although Trio populations in Suriname reside in the adjacent Guiana Highlands and not in the Amazon Basin proper, Greater Amazonia is generally considered to be inclusive of the Guiana Shield region [27].

Although basic subsistence activities persist largely unchanged, missionaries and other forces of acculturation have rapidly and profoundly impacted Trio culture. Even as early as 1963, traditional dances, festivals, and oral traditions had been abandoned through the strong dissuasion of American evangelical missionaries [24]. Over the past decade, some of these practices have been readopted - albeit in a modified form - in the absence of a strong missionary presence.

Medical knowledge and ability is specialized in Trio society to the shaman (*pijai*), who is principally responsible in this capacity for communication with the supernatural world [17,24,28]. Healing ceremonies take place in a small cabin (*mīnë*) constructed for the purpose, where a shaman would sometimes be accompanied by an assistant or apprentice [29]. Prior to contact, the use of tobacco was central in healing ceremonies as smoke was thought to nourish the spirits kept within the shamanic rattle (*maraka*) [24].

The Trio perception of health (*kurerëke*) and disease (*esenë*) is broad, encompassing etiologic concepts that are neither "physiologic" nor fit well within our own biomedical paradigm. The consumption of certain taboo animals, for example, may affect the strength (*kaarimë*) and characteristics of an individual [30]. Menstrual fluid is viewed as extremely polluting to the spirit [24]. The Trio, however, perceive some conditions as natural processes, not solely a consequence of social or cosmic disturbance [17]. Distinction is made between diseases of spiritual origin and those of non-spiritual origin, as well as between endemic diseases from those introduced through contact with foreigners, either non-indigenous (*pananakiri*) or semi-indigenous Maroon groups (*mekoro*) [17]. A similar duality in causation is also maintained among Kayapó and Tsimane' healers [14,22]. Although Trio shamans are widely accepted to have the capacity to incite illness, their influence, through the summoning of evil spirits (*wiripë*), can also act to mediate the course and outcome of natural illness [17]. Rare disease conditions, or particularly those refractory to conventional treatment, are often attributed to the work of shamans [17].

Botanical species from the surrounding rainforest biota are extensively utilized by Trio healers as therapeutics in addition to a few reported uses of fungi and arthropods [28]. In Trio society, knowledge of medicinal plants is predominately specialized to the *pijai *(shaman), although other individuals may have a limited general knowledge of botanical remedies [28]. Three extensive surveys of Trio medical ethnobotany have been conducted among populations on both sides of the Suriname-Brazilian border. The inventories, developed through interviews of Trio informants, support a prodigious medicinal plant knowledge. Frikel and Cavalcante in 1973 describe 171 plant species used for medicinal purposes by Brazilian Tiriyó [31]. The ethnobotany of the Suriname Trio was investigated from 1982-1986 by Plotkin, who reported 318 species of plants utilized by the Trios for medicinal purposes as well as other biodynamic uses such as arrow poisons and fish toxins [28]. A comparative investigation of Trio ethnoecology was conducted by Hoffman in the village of Kwamalasamutu contemporaneously at the latter end of the present study [32].

The eradication of shamanism as an institution of "witchcraft" was central to efforts of missionary activities in many Amazonian societies [33]. The use of tobacco, rattles, and chants in Trio healing ceremonies, a hallmark of Carib tribes, disappeared from practice [34]. Medicinal plant use, viewed by missionaries as compatible to Christian faith, was deemed acceptable but not strongly condoned. Concerning the present state of the spiritual aspects of Trio shamanism, Rivière commented in 1994 that the "old ideas have not disappeared" [35]. Anecdotal evidence reported by Herndon in 2004 attests to the vitality and strong undercurrents of shamanistic beliefs among the Suriname Trio that resisted proselytization [17].

In July 2000, a traditional medicine clinic (*Katamïimë Ëpipakoro*) was established in the village of Kwamalasamutu as part of a joint health promotion initiative of the Trio communities, the regional medical care provider to the Suriname interior (Medische Zending Suriname), and a biocultural conservation NGO based in Arlington, Virginia (Amazon Conservation Team). Elder Trio healers, widely recognized throughout the community as *pijai *(shamans), autonomously operate and direct all aspects of the traditional clinic's operation. Each clinic structure provides a facility for elder shamans to practice as well as allows for apprentices, who serve as clinic assistants, to directly observe the shamans actively practicing their medicine. Shamans in each village rotate practice in the clinic daily within an established schedule. Patient visits to the traditional medicine clinic are elective and without fee. In August 2001, a second traditional medicine clinic (*Kaapi Ëpipakoro*) was established in the Trio village of Përëre Tëpu, approximately 150 km to the east of the village of Kwamalasamutu.

## Methods

This study was designed and undertaken with the full consent and participation of the Trio communities, healers and traditional medicine clinic staff in both project sites. Institutional review board approval was obtained from academic affiliates (Yale University School of Medicine [New Haven, Connecticut, USA], Brigham & Women's Hospital, Harvard Medical School [Boston, Massachusetts, USA]) of the principal investigator (CH).

Traditional clinic staff workers, younger apprentices with basic literacy skills in a written articulation of the oral Trio language obtained through governmental schooling, were trained prior to the opening of each clinic to complete a printed record form (Additional file 1) at the direction of the elder Trio shamans for every patient that sought consultation and/or treatment at the clinic. The record form, translated into the Trio language with assistance of a linguistic specialist (Sérgio Meira, Ph.D., Museu Paraense Emílio Goeldi, Belém, Brazil), documents patient code number, identity of shaman, reason for visit, and treatment provided.

Each patient was assigned a code that was prepared by the Trio clinic workers from a government census list containing the name, gender and date of birth of the entire village population. Ages of the adult Trio born prior to historical records had been estimated at the time of contact through physical examination and through correlation of life histories with discrete, readily identifiable events (such as arrival of early expeditions).

The study period was from August 2000 to August 2004 in Kwamalasamutu and August 2001 to August 2004 in Përëre Tëpu. The clinic records were periodically collected from each project site and entered into an Excel^® ^database. Quantitative descriptive analysis of the clinic databases was performed in SPSS^® ^at the conclusion of the study.

An inventory of disease conditions documented in clinic records was generated from the database. A cross-cultural comparative methodological approach was utilized to articulate descriptions of Trio disease conditions as they are perceived by the indigenous healers [36]. Conversation was initiated in the Sranan Tongo and Dutch languages with the younger Trio clinic assistants, who in turn translated our inquiries into Trio for the elder shamans. A generative methodology was employed in which formal questioning was held to a minimum and the most general formulations were utilized. Representative statements included 'How do you recognize *ariminaimë *(electric eel disease)?'. To probe distinctions between apparently similar concepts, the informants were asked such questions as 'How is *otono *(upper respiratory tract infection) different from *iropï *(pneumonia)?' A photographic clinical reference was sometimes utilized to facilitate identification and assist in distinguishing between recorded dermatologic concepts [37]. Translations were cross-checked for accuracy and consensus of content on at a minimum of three field visits.

The development of valid comparisons of indigenous disease conditions utilizing biomedical terminology can be challenging as even the simplest disease concepts in biomedicine may comprise complex constructs of pathologic, physiologic, and structural ideas [38]. Careful attention was applied in the semi-structured interviews to delineate boundaries of Trio disease conditions, minimize ethnocentric bias in formulations, and avoid introduction of non-indigenous concepts. The extent of overlap between indigenous and biomedical disease conditions can be difficult to precisely discern. In our methodology, we refrained as much as possible from establishing direct equivalence between Trio and biomedical disease concepts except in discrete cases where clearly indicated, preferring to present in our descriptions the symptomatology and disease associations as reported by tribal healers, with postulated correlation, if any, to biomedical concepts. Cross-cultural comparative studies have been criticized by medical anthropologists for forcing "rich, complex ethnographic data into artificial categories" [36]. Abridged translations of Trio disease conditions are included within figures as well as other sections of this paper. These are approximate articulations provided for reference only and do not necessarily intend to imply complete equivalence between disease conditions.

Hierarchal distinction between disease symptoms and processes (e.g., abdominal pain versus appendicitis) in the concept understood within the biomedical system was deferred as its cross-cultural applicability to Trio ethnomedicine is unclear. In addition, although some Trio disease conditions may literally be translated as a single symptom, these disease constructs do not represent an isolated symptom, but rather a well-recognized disease complex named for a hallmark symptom. *Ere nakuikan*, for example, literally "liver pain", is characterized by Trio healers as a complex of mid-gastric radiating to right upper quadrant pain, jaundice, weight loss, and enlarged liver suggestive of hepatitis, a common endemic disease of the Amazonia. Similarly, *kananaman*, translates as "yellowing" or "jaundiced", however, the concept, the traditional Trio term for malaria, generally refers to the widely-recognized disease complex of alternating high fevers, headache, and scleral icterus. For the purposes of this study, all recorded reasons for visits to the traditional medicine clinic were considered broadly as "disease conditions" without further hierarchical delineation.

In our analysis, disease conditions were secondarily categorized into biomedical systems according to the principal manifestation of the disease. Although not validated in our study methodology as Trio disease taxonomy, this classification system permits useful adjunct comparative analysis of the large number of indigenous disease conditions. Classification of illness in a comparable manner has been described among other South American indigenous groups, including the Warao and Matsigenka [10,12]. Assignments to system were determined by site of principal symptom manifestation and not by presumed etiology. For example, *marareja *(malaria), an infectious and hematological disease in our understanding, was classified as systemic disease corresponding to its predominant symptom of fever. Although significant potential exists for overlap (*i.e*., a respiratory disease may also have associated systemic or other disease system manifestations), most disease conditions were readily categorized into one primary system.

As a supplement to our study of disease concepts, Trio shamans were interviewed to produce a comprehensive survey of indigenous anatomical nomenclature. A linguistic study of Trio body party terminology, with focus on morphological, syntactical, and semantic aspects, was conducted independently by Meira and published subsequent to the completion and initial reporting of our fieldwork [17,39]. In our methodology, Trio healers were asked to point out and identify structures on basic figures from an anatomical atlas, representing the whole body (anterior and posterior), skeletal/bony structures, facial features, as well as internal and reproductive anatomy [40]. Elicited nomenclature for identified structures was cross-checked on at least three subsequent visits for consensus validation as well as to minimize inclusion of nonce terminology.

Quantitative analysis of medicinal plant utilization in regards to use-consensus and ecological preferences in botanical family, plant form, and collection zone will be examined in separate publications. Specific botanical identity of medicinal plants will not be disseminated in any publication resulting from this study to protect indigenous intellectual property rights in accordance of the wishes of the investigators and participating communities.

## Results

18,199 patient visits were recorded at the traditional medicine clinic (*Katamïimë Ëpipakoro*) in the village of Kwamalasamutu (KM) during the four-year interval from August 2000 to August 2004. In the smaller village of Përëre Tëpu (PT), 2,138 patient visits to the traditional medicine clinic (*Kaapi Ëpipakoro*) were documented between August 2001 and August 2004.

**Figure 2:**
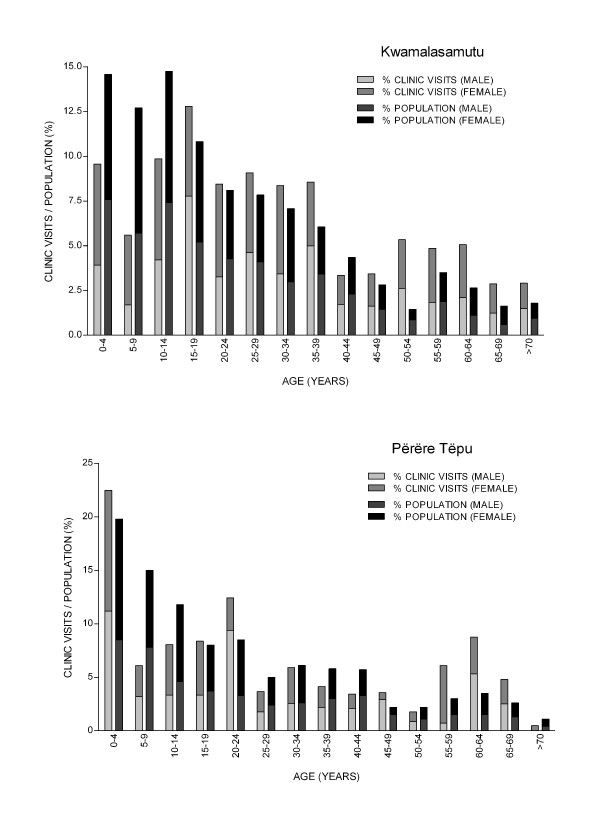
**Demographics of traditional medicine clinic utilization**.

Demographic characteristics of clinic utilization are summarized in Table 1 and Figure 2 in reference to the respective village census population. Age and gender information was not available for patients in 12.3% (2241) and 0.9% (37) of clinic records for KM and PT, respectively, a deficit in Kwamalasamutu attributable in part to clinic utilization by visiting Trio from the village of Missão Tiriyo, from which census data is unavailable. These records were excluded from the demographic sub-analysis.

**Table 1:** Demographics and patient utilization of traditional medicine clinics.

	**Kwamalasamutu (KM)**		**Përëre Tëpu (PT)**	
**Location**	2° 19' 60 N / 56° 46' 60 W Sipaliwini river Sipaliwini District, Suriname		3° 10' 00 N / 53° 43' 00 W Tapanahoni river Sipaliwini District, Suriname	

**Population census**^1^	1174		503	

**Study interval**	8/2000 - 8/2004		8/2001 - 8/2004	

**Total clinic visits**	18,199		2138	

**Clinic visits per month**^2^	368.9 ± 33.8 [47-1148]		60.0 ± 37.8 [11-183]	

**Total patients**	765		180	

**Clinic visits per patient**^2^	21.1 ± 26.1 [1-167]		11.7 ± 9.6 [1-63]	

**Patient age at clinic visits**^3^	30.3 ± 19.8 [.02-80.6]		27.3 ± 22.0 [0.3-70.1]	

**Clinic visits per gender**	M 7475 [46.6%]	F 8566 [53.4%]	M 1079 [51.4%]	F 1022 [48.9%]

**Clinic visits per disease concept**^2^	225 ± 53.0 [1-2629]		49.8 ± 10.2 [4-291]	

**Clinic visits per shaman and shaman age at start of study interval**	KM shaman 1 (69.4 years)	10,354 [56.9%]	PT shaman 1 (66.7 years)	1255 [58.7%]
	
	KM shaman 2 (63.6 years)	3708 [20.4%]	PT shaman 2 (66.5 years)	766 [35.8%]
	
	KM shaman 3 (53.8 years)	864 [4.7%]	PT shaman 3 (70.4 years)	117 [5.5%]
	
	KM shaman 4 (51.9 years)	679 [3.7%]		
	
	KM shaman 5 (50.0 years)	571 [3.1%]		
	
	Not recorded	2023 [11.1%]		

**Shaman age at clinic visits**^3^	68.0 ± .04 [51.6-73.5]		68.2 ± .02 [66.6-71.7]	

^1^2004 census, an approximate cross-sectional assessment as the population dynamics of Suriname Trio villages are fluid.

^2^Values expressed as mean ± STD [range]

^3^Values expressed in years, mean ± STD [range]

The mean age of practicing shamans at time of clinic visits was comparable between the villages of Kwamalasamutu and Përëre Tëpu at 68.0 and 68.2 years, respectively. These values, derived from dates of birth assigned at the time of initial contact, comprise only approximations of natural age. The differential distribution of clinic visits per shaman (Table 1) is not directly attributable to patient selection as shamans rotated on an established schedule, determined by the healers in part on seniority as well as availability in the village. Two of the healers (Shamans 4 and 5) in Kwamalasamutu did not begin practice in the traditional medicine clinic until the final twelve months of the study interval. One shaman (Shaman 3) in Përëre Tëpu only practiced in the clinic while he was visiting the village for a short residence of three months.

**Figure 3:**
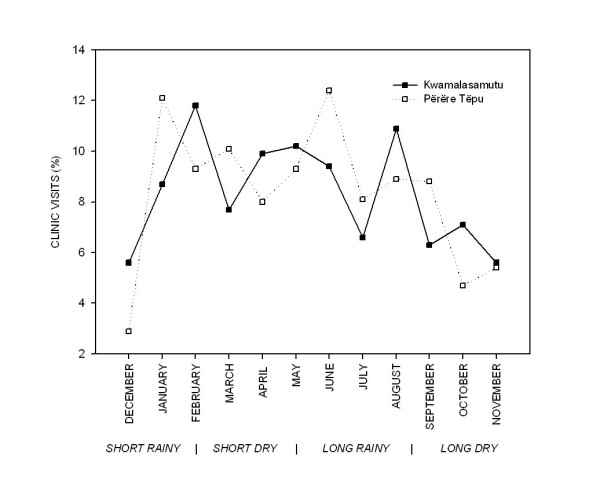
**Seasonal trends in traditional medicine clinic utilization**.

Utilization in both villages was comparable across major climate seasons (Figure 3), with bimodal peaks during the long rainy season (May to mid-August) and at the transition from the short rainy to the short dry season (January to February). The highest frequency of clinic visits in both villages occurred during the long rainy season (37.5% KM vs. 38.7% PT), months during which morbidities are typically more prevalent in lowland Neotropical communities.

Seventy-five disease conditions were recorded by Trio healers, ranging from generalized (e.g., fever [*këike*]) to specific and rare medical conditions (e.g., Bell's palsy [*ehpijanejan*]). Full cross-cultural translations of disease conditions are presented in Tables 2, 3, 4, 5. A combination of two or three discrete disease conditions, i.e. *eikeke *+ *iputupë nakuikan *(wound + headache), was documented in 15.3% (483) KM and 5.1% (110) PT of clinic visit records. 1.3% (237) KM and 0.9% (20) PT of disease condition entries were not interpretable or incompletely filled in clinic records, necessitating exclusion from the sub-analysis of disease conditions.

**Table 2:** Dermatologic Disease Conditions Treated by Shamans of Kwamalasamutu and Përëre Tëpu.

**Trio Disease Concept**	**Diagnostic symptom(s) and descriptive field notes**
**Dermatologic:**	

*Awë*	Acne. *Kënepoko *is a specific term for pus-filled comedones. Trio shamans associated *awë *with ingestion of particular foods, for example, the roe (*kana iimo*) of *aymara *(*Hoplias macrophthalmus*) and *pasina *(*Myleus pacu*) fish.

*Eikeke*	General term for wound, including insect bites. The scar resulting from a wound is termed *tikoroje*. *Eikeke niketan *refers to a wound that has a superimposed infection.

*Iikë*	Myiasis (Botfly, *Dermatobia hominis*). Botanical treatments are directly applied to lesion to kill larvae; dead larvae are subsequently extracted.

*Ijatuhpë*	Old burn wounds. A fresh burn is described as having warmth (*atuma*), erythema (*tamiren*), and intense tenderness (*nakuikan*) at the site, which resolves over a period of several days with the development of white granulation tissue.

*Ikurutato*	Contact dermatitis, described as rash and pruritus occurring after exposure to irritants, e.g. urticating hairs of a tarantula (*moi*).

*Iroitesikae*	Deattachment of toenail.

*Juwi*	Furuncle. Superficial infection of skin around hair follicles. Shamans place medicine into the lesion and squeeze out residual pus.

*Kaasa*	Leishmaniasis, a condition attributed by Trio to an insect bite although they are non-specific to insect identity. Trio note that *kaasa *is frequently acquired in the course of hunting trips to particular swamp regions. Sandfly and mosquito are known to the Trio, respectfully, as *mapyiakë *and *makë*.

*Manahai*	Mastitis.

*Moto ipuhpo*	Literally "*Ascaris *of the feet", *moto ipuhpo *refers to the cutaneous larva migrans of the hookworm, presumably *Necator americanus*. According to Trio shamans, *moto ipuhpo *infestations may cause anemia (*imununna*).

*Onoe*	Carbuncle. Deep-seated, walled-off pyogenic collection. Initial treatment for *onoe *is directed toward mechanically opening the abscess.

*Osi*	Superficial dermatomycosis.

*Paikarakahpë*	Lichenification of skin secondary to chronic inflammation, most commonly appearing on the peri-oral, penile and vulvar regions.

*Pireimë*	Infestation of harvest mites (*pirë*), causing severe pruritius (*pirëkëne*) of affected areas. Trio state that although scratching provides temporary relief, chronic scratching may result in *karakalali*, a dermatologic condition characterized by discomfort, erythema, fissuring of the skin with exudates.

*Pitotoimë*	Disease concept characterized by the appearance of water-filled vesicles, which initially develop on the face and subsequently spread to remainder of body. The condition, possibly consistent with Varicella, is described by Trio elders as particularly contagious in children but older individuals may acquire it they have not previously had the disease. *Pitotoimë *is reported to be rare with few recent cases and is reported to be an endemic condition predating non-indigenous contact. Measles, known as *kurukuimë*, is considered an introduced disease and was associated with significant mortality during an outbreak among the Trio in 1971.

*Pupara*	Fissure of the heel.

*Tëekae*	Bite wounds. Animal bites treated by Trio healers during study interval include canine (*kaikui*), piranaha (*pëne*), and snake (*ëkëi*).

*Tesowakae*	Superficial scrape wound.

*Tijase*	General term inclusive of all burn wounds. Burns among Trio adults commonly occur through the preparation of hot cassiri (*kurula*), a fermented beverage derived from masticated cassava.

*Tikonkae*	Injuries inflicted from mechanical impact of sharp foreign objects e.g. splinters (*ikonkahpe*) or nails. Stingray (*spari*) stings are recorded as *spari tikonkae*. *Emëinë *refers to commonly encountered puncture injuries of the sole from the spines of the awara palm tree (*amana*).

*Tikuruje*	Pruritic dermatitis affecting the inguinal and gluteal regions that occurs in all age groups, described as commonly encountered after wearing unchanged clothing for a prolonged period.

*Tiroikae*	Deep scrape wound.

*Tiwëhtahkëse*	Cut wounds, such as those inflicted by wood or machete (*kasiparaja*).

**Table 3:** Gastrointestinal and Genitourinary Disease Conditions Treated by Shamans of Kwamalasamutu and Përëre Tëpu.

**Trio Disease Concept**	**Diagnostic symptom(s) and descriptive field notes**
**Gastrointestinal:**	

*Ere nakuikan*	Liver pain; associated with yellowing of eyes (*kanamë enu*), nausea (*ninujaman*), emesis (*yiwenatae*), weakness (*arerenna*), and dark-tinged urine. Trio healers describe pain distribution ranging from epigastrium extending to the right upper quadrant. Hepatomegaly (*ererimpë toje*) is noted on examination. Clinical correlation is suggestive with hepatitis, a common endemic disease of lowland Amazonia.

*Eta nakuikan*	Spleen pain; a disease concept characterized by left upper quadrant abdominal pain and an enlarged spleen on examination. Splenomegaly is associated by Trio healers with chronic malaria (*kananaman*).

*Iwaku nakuikan*	Abdominal pain, non-specific. *Niwapahhan *refers to tenesmus, electric sensations in the abdomen compared by Trio healers to the shock of an electric eel (*arimina*), often preceding profuse episodes of watery diarrhea.

*Iwaku toje*	Swollen abdomen, recognized by Trio shamans as pathologic, yet of uncertain etiology, attributed by one shaman as a result of "too much air in abdomen".

*Manimanikë*	Pinworm (*Enterobius vermicularis*), common among children and associated with perianal pruritus.

*Moto*	*Ascaris lumbricoides*. According to Trio, *Ascaris *worms "eat when you eat" and heavy infestations are believed to lead to failure to gain weight and abdominal pain secondary to obstruction. *Amepa *refers to infection from the dog tapeworm *Dipylidium caninum*, in which segments of the nematode are noted in stools.

*Niputan*	Watery diarrhea.

*Niputan munune*	Dysentery. Watery diarrhea with blood (*munu*) and/or mucus (*asinokato*)

*Urutupë nakuikan*	Dyspepsia is referred to as stomach pain (*urutupë nakuikan*), described by Trio as burning inside the stomach (*urutupë atuma awe*), which may be exacerbated by dietary intake of certain foods, for example hot peppers (*pëmëi*).

*Waku*	General term for diarrhea. Alternative term for loose stool is *tiputae. Iweti *is the Trio term for normal feces; constipation is *koekaewa*.

**Genitourinary:**	

*Ejamori nakuikan*	Kidney pain. Flank pain; associated with dark urine (*suku*), commented by Trio shamans to sometimes occur with severe malaria (*marareja*).

*Imone kureta*	Literally translated "evil uterus", the disease concept was applied to vaginal bleeding in elder women (*nosi*).

*Imone nakuikan*	Dysmenorrhea, literally translated "uterine pain".

*Imone toje*	Swollen uterus with persistent bleeding not in association with pregnancy (*minome*). Trio healers stated if untreated may progress to death.

*Isuku nakuikan*	Dysuria. *Isuku kureta*, literally translated 'bad bladder', this term is applied when gross hematuria is present. The Trio attribute the condition to sexual intercourse with other non-marital partners or from trauma. Gonorrhea is referred to as *ëriime *and is stated to be a non-endemic disease condition.

*Nimuntan*	Menses. Heavy and prolonged menses with associated passage of large clots (*munu tëpu*, literally "blood stones") is referred to as *iyeta nimutan*. In Trio cosmology, menstruation has associations with the origins of the Moon (*Nunnë*). Although menstrual fluid is considered extremely polluting to the spirit, menstruation is believed a natural process necessary for fertility. Abnormal uterine bleeding is considered as a disease state (*esenë*).

**Table 4:** ENT/Opthamalogic, Musculoskeletal and Neurological/Psychiatric Disease Conditions Treated by Shamans of Kwamalasamutu and Përëre Tëpu.

**Trio Disease Concept**	**Diagnostic symptom(s) and descriptive field notes**
**ENT/Ophthalmologic:**	

*Enu nakuikan*	Eye pain, non-specific. Trio healers describe a rare condition called *enu kureta*, which presents with very severe eye pain of an acute and severe nature.

*Enunkë*	Conjunctivitis, characterized by irritation (*pirëkëne*), injection (*tamiren*) and purulent drainage (*ikumuru*) of the eye. Cataracts and hordeolum are known, respectively, as *tëmakapuruja *and *empilo*.

*Ije nakuikan*	Tooth pain. Trio apply the soft wood of the *lapa lapa *tree to the gums, which swells to help dislodge tooth root prior to extraction.

*Ipana nakuikan*	Otitis. Extracts of botanical treatments are taken systemically and applied directly into the ear.

*Mitaikë*	Aphthous ulcer (canker sores). Oral mucosal lesions described as small, round, and painful ulcerations. Genital herpes also are referred to as *mitaikë*. Oral candidiasis, sometimes encountered among suckling infants, is known as *susu mitaikë*, literally "milk *mitaikë*". *Mitakapiru *refers to angular stomatitis (cheilitis) at the corners of the lips secondary, in biomedical understanding, to riboflavin (Vitamin B2) deficiency.

*Timirise*	Fishbone lodged in throat. *Yenatëka *refers to coughing after passage of food or drink through the airway.

**Muscloskeletal:**	

*Akiakinë*	Arthritis. Focal pain and swelling in joints seen in elderly, affecting different joints but principally knees, cervical and metacarpal joints, exacerbated after working hard and in cold hours of the morning.

*Iponi epine nakuikan*	Pain and mild swelling at level of umbilicus such as following trauma. *Taririme *is a subcutaneous hematoma (bruise) such as resulting from blunt trauma.

*Ijetipë nakuikan*	Pain attributed to joints and bones, including lower back (*aharapa*), hip (*ekun*), neck (*ipimi*), heel (*itëpu*), knee (*iwerena*), caused by trauma and exertion.

*Kukutuma*	Rheumatism. *Kukutuma *refers to diffuse whole body pain, effecting both young and old alike, but is also applied to myalgias experienced following a long expedition or accompanying an illness.

*Teekitonëe*	Wrist sprain, in which joints are twisted (*nepanemenjan*) in a motion similar to the spooling of cotton. *Temahinkayakae *is specific for ankle sprain.

*Tepahhai*	Bone fracture.

**Neurological/Psychiatric:**	

*Ahhëtepë nakuikan*	Post-operative pain from surgical procedures, e.g. tubal sterilization. Trio report that no surgical procedures were performed prior to contact beyond opening of abscesses (*onoe*), perforation of earlobe and lower lip, and cutting of the umbilical cord.

*Iputupë kureta*	Psychosis. Literally translated 'bad head', the Trio describe examples of behavior associated with *iputupë kureta *as talking to people who are not present and ingestion of dog feces. Patients are described as functioning poorly in their daily lives (unable to hunt or fish) and the disease is thought not to respond well treatment.

*Iputupë nakuikan*	Headache. Trio elaborate no distinction between types of headache.

*Ehpijanejan*	Facial nerve palsy [Bell's]. Trio healers state that *ehpijanejan *occurs among all age groups but is very rare and attributable to the curse of a *pijai*.

*Nenmerejan*	Vertigo, described as sensation of the head and eyes spinning.

*Nipirërujan*	Focal paralysis of an extremity.

*Sinsinman*	Paresthesias. 'Pins and needles' sensation occurring in absence of rash or other dermatological manifestations. The Trio compare *sinsinman*, which may affect the entire body, to tingling that occurs in the lower extremity after prolonged sitting (referred commonly in our culture as 'foot fell asleep'), or to the sensation of a spider crawling on the skin. *Sinsinman *is reported to be often experienced in the later stages of malaria following administration of anti-malarial medications.

**Table 5:** Cardiorespiratory and Systemic Disease Conditions Treated by Shamans of Kwamalasamutu and Përëre Tëpu.

**Trio Disease Concept**	**Diagnostic symptom(s) and descriptive field notes**
**Cardiorespiratory:**	

*Akuruku*	Costrochronditis. Stabbing pain (*nikonkan*) in the thorax on inspiration, such as following from a prolonged respiratory illness.

*Ewanë nakuikan*	Midsternal pain attributed to the heart (*ewanë*), occurs at rest, associated with palpations, can be fatal, "when it hurts you can die"

*Iropï*	*Iropï *is the Trio anatomical term for chest but, in context of a disease concept, refers to a complex of thoracic pain accompanied by coughing (*tontonkato*), labored breathing (*nerepakejan*) and high fever (*këike*), suggestive of pneumonia. Trio shamans state that *iropï *can sometimes prove fatal and is noted to occur as a complication of colds (*otono*). The modifier *tïkëtae *(*iropïtao tïkëtae*) describes the production of a copious, foul-smelling, yellow-green sputum.

*Otono*	Common cold, upper respiratory tract infection. *Otono *refers a well-recognized complex of rhinorrhea, cough (*tontonkato*), and pharyngitis.

*Otonoimë*	*Otonoimë *describes a disease concept, highly suggestive of pulmonary tuberculosis, of a long-standing cough, hemoptysis and night fevers that is reportedly progressively fatal if untreated.

**Systemic:**	

*Amiima*	Fatigue.

*Ariminaimë*	Electric eel (*arimina*) disease, described electricity going through your body, compared by Trio healers as similar to the sensation experienced on tapping the ulnar nerve at the exposed point on the medical epicondyle. *Ariminaimë *is distinct from epilepsy (*newatanjan*), a well recognized but extremely rare disease condition for which there have been no reported cases in recent years.

*Arerenna*	Weakness accompanying chronic illness.

*Ijemira*	Anorexia in the context of illness.

*Imununna*	Anemia, a disease state characterized by loss of conjunctival rubor (*ëmperu tikoroje*), labored breathing and fatigability (*amiima*).

*Ipun awë atuma*	Warm flushing of the body with associated diaphoresis (*niritan*) accompanying illness.

*Kankë*	Trio adaption of the biomedical term cancer. Cervical carcinoma (*epah sesereimë*) is known to the Trio, the etiology of which was attributed by one elder shaman to a devil spirit arising from the penis.

*Këike*	Fever. *Këike *is characterized by the Trio as a high grade fever in contrast to *sunaime*, an early developing low grade fever.

*Marareja*	Trio adaption of the Dutch biomedical term malaria. *Kananaman*, the traditional Trio term for malaria, a disease concept recognized by cyclic high fevers (*këike*), bilious emesis (*yiwenatae*), headache (*iputupë nakuikan*) as well as corporal and/or scleral icterus (*kanamë enu*).

*Nikëipainjan*	Malaise. *Nikëipainjan *refers to prodromal symptoms of a developing illness including rigors (*nitëtëpainjan*) and myalgias (*kukutuma*).

*Tikurike*	Lymphadenopathy, often noted in the presence of sickness. Trio healers identify regional distinctions in lymphadenopathy with suffix -*piri*: *ematapiri (*inguinal), *ijatapiri (*axillary), *enapiri *(cervical-submandibular) and *ipanapiri *(posterior auricular) lymphadenopathy

*Tinome ipun*	Cold sensation in the body, commented to accompany severe illness such as cancer (*kankë*).

**Figure 4:**
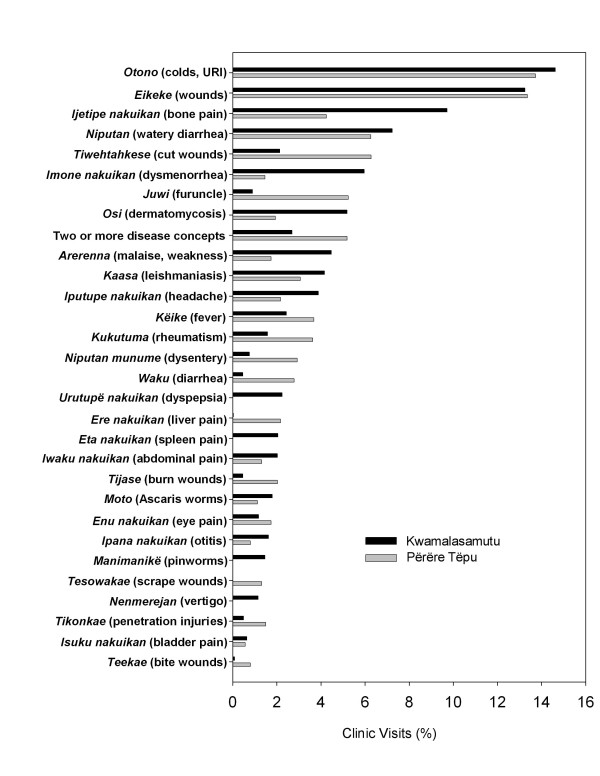
**A comparative ranking of the most frequently treated disease conditions by Trio shamans based on patient visits to the traditional medicine clinics in Kwamalasamutu and Përëre Tëpu**.

**Figure 5:**
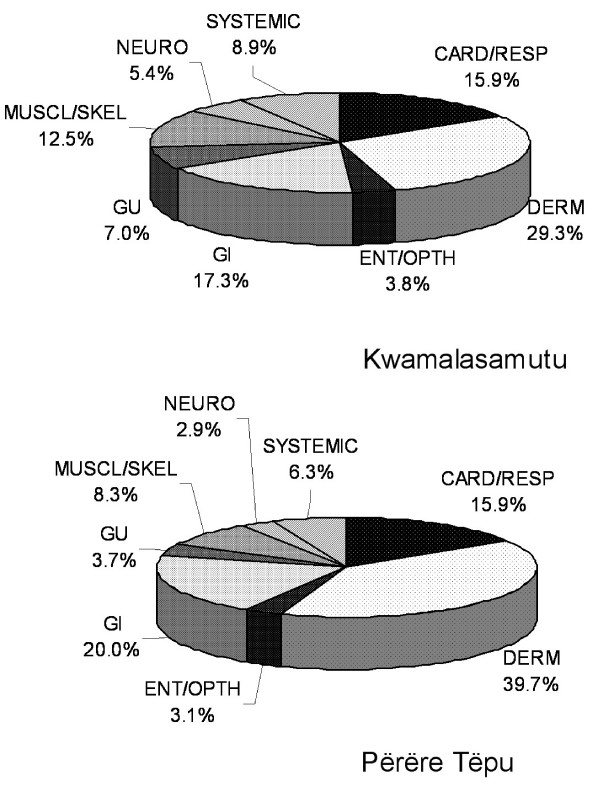
**Disease treatment by biomedical system**.

Figure 4 demonstrates the thirty most frequently treated disease conditions at both project sites, indicating a comparable spectrum of disease presentation by both populations. 45.3% (34/75) of disease conditions were treated at both villages; the remainder conditions were infrequent or rare and generally only represented within the larger population of Kwamalasamutu. Figure 5 compares clinic utilization by biomedical disease system. The greatest representation of disease and patient visits comprise morbidities common to lowland tropical regions including dermatologic conditions, gastrointestinal afflictions, and respiratory contagions.

In their responses, Trio informants present highly detailed and specific descriptions of disease characteristics and associated symptomatology. Among dermatologic conditions, for example, Trio healers discriminate between subtle aspects of the natural healing response as well as clinical features of acute versus chronic inflammation. In their descriptions, Trio shamans also comment on disease associations and responsiveness to therapy, often demonstrating a remarkable insight into the natural history of disease processes. Trio shamans correlate splenomegaly of *eta nakuikan *with long-standing malaria, the cause of hyperreactive malarial splenomegaly. Anemia (*imununna*), diagnosed through examination of the conjunctiva, is attributed to hookworm (*moto ipuhpo*, a term literally translated as "*Ascaris *[a large, intestinal worm] of the feet", in reference to the characteristic cutaneous larva migrans). Postmenopausal bleeding in women is identified as ominous, referred to as *imone kureta *(evil uterus). Psychosis (*iputupë kureta*) is considered generally refractory to treatment.

The observations of our study support a strong degree of convergence in shared perception between Trio ethnomedical and Western biomedical systems, as many Trio disease conditions corresponded, at least approximately, if not quite closely, to disease constructs within our own medical system. Among respiratory conditions, for example, Trio shamans identified three distinct conditions corresponding closely to our concepts of upper respiratory tract infection (*otono*), pneumonia (*iropï*) and tuberculosis (*otonoimë*). Pneumonia (*iropï*) is reported to sometimes occur as a complication of upper respiratory infections (*otono*). *Otonoimë *refers to a disease complex of long-standing cough, hemoptysis and night fevers that is reportedly progressively fatal if untreated. Surveys in this past decade indicate a relatively high prevalence of active and latent tuberculosis among Trio in Kwamalasamutu [41]. A few disease conditions, such as *ariminaimë*, encountered in the course of the fieldwork were culturally-specific concepts that had no readily identifiable direct biomedical correlate.

**Figure 6:**
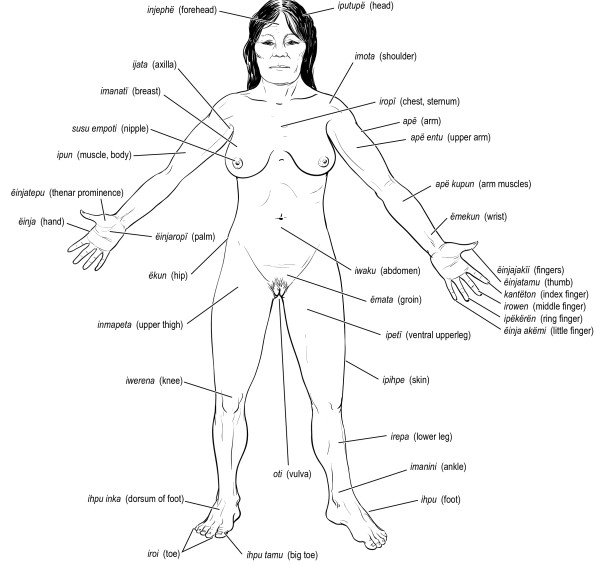
**Major anatomic structures recognized by Trio shamans - anterior full body**.

**Figure 7:**
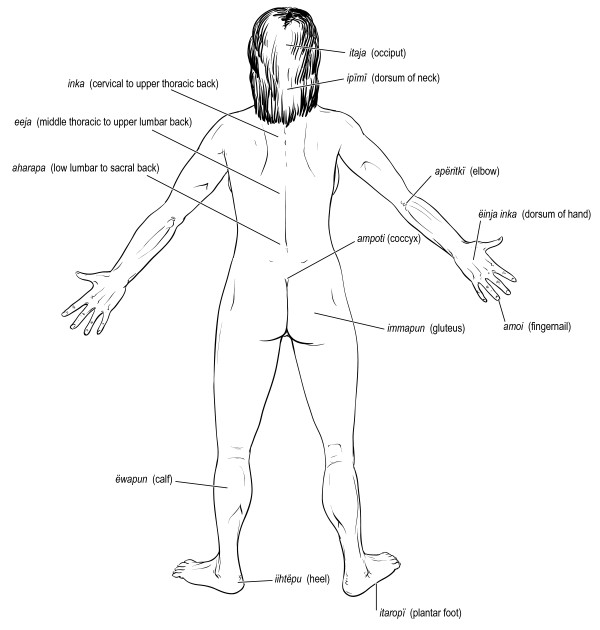
**Major anatomic structures recognized by Trio shamans - posterior full body**.

**Figure 8:**
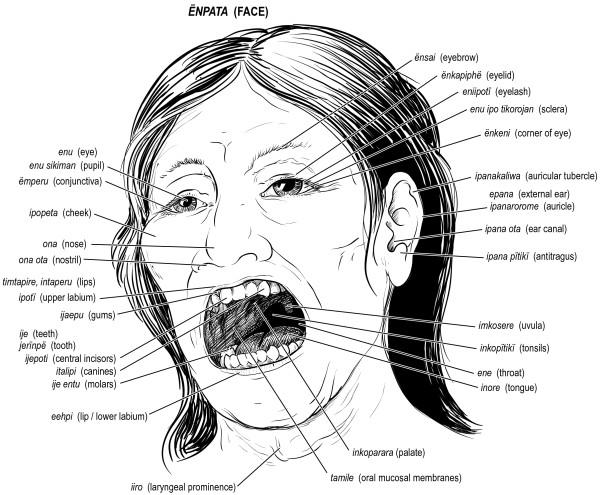
**Major anatomic structures recognized by Trio shamans - face**.

**Figure 9:**
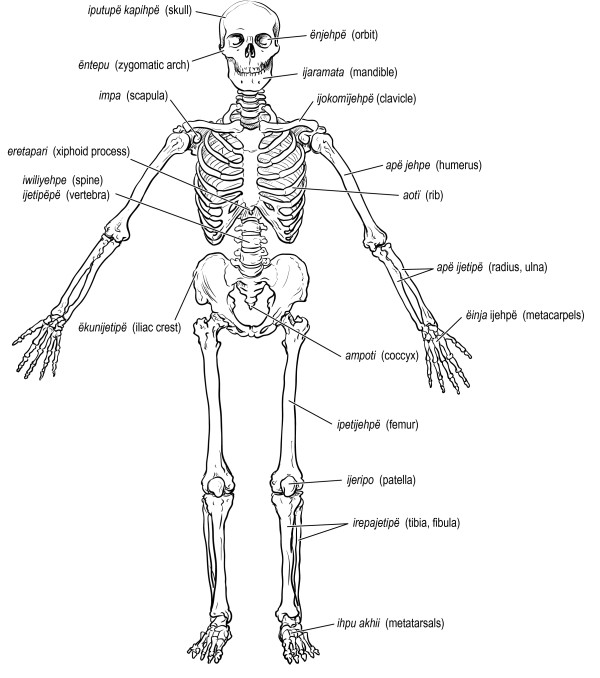
**Major anatomic structures recognized by Trio shamans - bony skeleton**.

**Figure 10:**
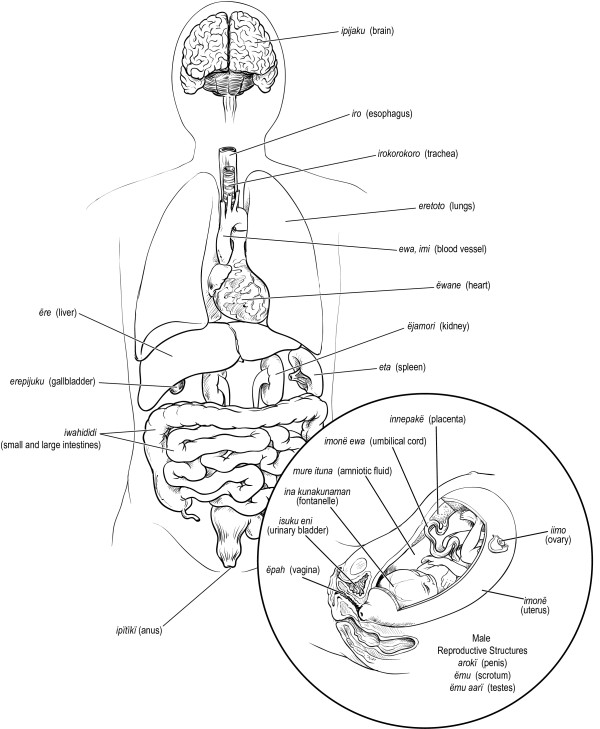
**Major anatomic structures recognized by Trio shamans - internal & reproductive organs**.

Figures 6, 7, 8, 9, 10 illustrate 127 anatomical structures recognized by Trio healers. All major internal organs and their anatomic relationships are identified by the Trio with exception of the pancreas. Trio shamans correlate regional distribution of abdominal pain with respective site of visceral organ involvement in a homologous manner as Western medical practitioners. The diagnostic role of physical findings on abdominal palpation can be inferred from the Trio association of *ere nakuikan *and *eta nakuikan *with hepatic and splenic organomegaly, respectively. Although the ovary is referred to as *iimo*, or egg, a morphological assignment rather than functional understanding is likely implicit; the Trio healers believe that conception arises from the interaction of sperm (*ikuru*) and uterine blood (*imone munu*) [42]. Sub-classification of levels of vertebral back as well as identification of major regional lymphatic groups is closely parallel within our own system.

## Discussion

In his 1975 *Science *article entitled 'The Need for an Ethnomedical Science', Fabrega advocates for the potential contributions of a discipline that examines how members of different cultures think about disease and organize themselves toward medical treatment [38]. He considers the reluctance of investigators to adopt a comparative approach to disease a biomedical ethnocentrism that fails to recognize the potentially valuable contribution of other cultures toward insights about disease and health. Others have followed to criticize medical anthropology for its emphasis on meaning and symbolism in health to the neglect of cross-cultural analysis in studies of non-Western medical systems [36]. Biomedical comparisons of native disease conditions can have important value in a diversity of applications, ranging from the screening of medicinal plants for bioactivity to health provision initiatives to indigenous communities [36,38,43]. The fundamental relevance of the study of disease to health and science, argued herein, is perhaps best expressed in a phrase from Cervantes' Don Quixote: the beginning of health lies in knowing the disease [44].

Full consideration of indigenous disease concepts is critical for the proper interpretation and evaluation of the therapeutic value of traditional treatments. Concepts of efficacy as perceived from within a culture (emic) may be different than those measured through the precepts of Western biomedicine (etic) [43,45-47]. In other words, traditional treatments can only be fairly judged for efficacy when considered through their own cultural standard. Medicinal plants, for example, may not strictly act as curative agents of a specific disease - the understanding most accessible from a biomedical perspective - but may also potentially alter the body-state and protect against negative spiritual elements [48,49]. The findings of our study additionally affirm the value of examination and precise translation of indigenous medical concepts within ethnobiologic studies. For example, a generalized translation 'rash' could be inappropriately applied to a number of discrete dermatologic conditions identified in Trio ethnomedicine, including dermatitis (*tikuruje*), dermatomycosis (*osi*), chronic inflammation (*paikarakahpë*), and contact dermatitis (*ikurutato*). The careful articulation of biomedical translations of indigenous disease concepts can be a challenging and meticulous process that requires a shared collective of experiential medical knowledge among investigators and traditional healers to properly discern boundaries of specific disease conditions.

The general reluctance of scientific investigators of Amazonian cultures to explore indigenous diagnostic concepts may have possibly contributed to a widely held perception that the medical understanding of so-called aboriginal healers is 'primitive' in comparison to their celebrated abilities, for example, as sophisticated empiric plant chemists [50,51]. Evidence exists from other Neotropical indigenous cultures to challenge this preconception. Peruvian highland groups, for example, as early as 18^th ^century applied the same term, *uta*, to both cutaneous leishmaniasis and the sandfly, an association was only postulated by Western epidemiologists as late as 1924 [52]. Extensive studies by Berlin et al. among the highland Maya of Chiapas have established that use of medicinal plants by Mayan healers is rationally guided by a sophisticated understanding of disease and symptomatology [53]. The results of our study support the presence of a comprehensive and highly formalized ethnomedical institution within the Trio culture. Our findings provide a foundation for further inquiry examining disease understanding and diagnostic methodology of Trio healers as well as more broadly point to the value for ethnomedical studies of other Amazonian indigenous cultures.

As disease in the context of a biological phenomenon is generally shared across the human species, significant potential exists for homology in disease recognition and understanding across cultures. Although only a paucity of ethnomedical studies of other Amazonian groups exist in the literature for comparison, disease conditions recorded by Trio shamans in our study are strikingly similar to those described among the Matsigenka of the Peruvian Amazon [10]. Trio and Matsigenka healers, for example, both associate spleen pain (*eta nakuikan *and *taratagantsi*, respectively) to long-standing malaria, a complication recognized as hyperreactive malarial splenomegaly in the biomedical system. Splenomegaly was a prominent physical finding in 91% of Trio children and adults examined in an early Suriname medical expedition in 1952 to the Trio of the upper Tapanahoni [54]. Interestingly, the Yanomamö of Venezuela appear to make a similar association between malaria and splenomegaly; 'spleen' and 'malaria' are both referred by the same Yanomami term (*hura*) [7]. The Wayãpi of French Guiana reportedly apply medicinal plants to swollen spleens [55].

The observations of our study additionally support a strong degree of convergence in shared perception between Trio ethnomedical and Western biomedical systems, as many Trio disease conditions corresponded, at least approximately, if not quite closely, to disease constructs within our own medical system. Although divergence between indigenous and biomedical understanding of illness is widely and appropriately cited in cultural studies, native concepts of disease causation may also have an underappreciated potential for convergence to biomedicine at least at some level. Matsigenka healers, for example, attribute acute gastrointestinal conditions, otitis, conjunctivitis, sexually transmitted disease, as well as various skin infections to the presence of thread-like, "so small as to be invisible" worms (*tsomiri*), a concept closely homologous to microbes within biomedical understanding [10]. An elder Trio shaman commented in our study that *epah sesereimë*, a rare disease condition highly correlative of cervical cancer, was caused by a "devil spirit from the penis". It is interesting to note that the germ theory of illness was not accepted into Western science and medical practice until well into the latter half of 19^th ^century [56]. Association of cervical cancer to an infectious agent, the human papillomavirus, was not established until 1976, although early epidemiologic studies suggested a pattern of sexual transmission [57].

Few comprehensive surveys of anatomical terminology in Amazonian cultures have been published. Chagnon, in his well-known ethnography of the Yanomamö, suggests a perspicacity of indigenous anatomical nomenclature, commenting that the Yanomami lexicon includes terminology for which there are no single word biomedical equivalent, e.g., "inside the elbow" and "back of the knee" [58]. The Trio have a corresponding term to the latter site, *karuteena*, commented by one informant as "a place where snakes like to bite" [39]. Despite the centrality of the body to existence and its universality as a physical constant across the human species, it is surprising how few comparative studies have examined the categorization and semantics of anatomical terminology of other cultures. In response to this deficit, the Max Planck Institute of Psycholinguistics sponsored in 2006 a remarkable series of cross-linguistic studies on the anatomical conceptualization of ten cultures worldwide, of which Meira's study of the Trio was included [59]. A standardized field guide and protocol for elicitation of body part terminology was additionally proposed [60]. Trio anatomical nomenclature independently elicited in our study is consistent with the terminology presented in Meira's linguistic analysis [39]. Our complete presentation of Trio internal and reproductive anatomical terminology represents an original contribution. The closest parallel to our findings in the scant literature of other Amerindian cultures is the celebrated anatomical knowledge of the Inuit of North America [61-63]. The familiarity of internal anatomical relationships of the Trio is likely derived, as thought in the case of the Inuit, from the comparative study of slaughtered large game mammals, although ritual dissection and removal of human organs was reputed to have been performed by Trio prior to contact [30].

Utilizing a novel methodology, we present the first study, to our knowledge, to investigate patterns of actual disease identification and treatment as recorded by healers of an Amazonian indigenous culture. The breadth of our study, involving analysis of over 20,000 patient visits throughout a period of four years, is without precedent in the literature and permits examination of a wide diversity of disease concepts, including comparatively rare and transient conditions, such as Bell's palsy and other neurological phenomena. Our study is limited, however, in its representation of Trio ethnomedicine as it is only inclusive of conditions presented by patients to the traditional medicine clinics for treatment. Several common ailments, notably conjunctivitis and sexually-acquired conditions, are conspicuously absent or underrepresented among diseases treated at traditional medicine clinics in both villages over the study interval. Although Trios have medicinal plants for these conditions, these infections respond rapidly to erythromycin drops or short-course antibiotics [28]. A similar phenomenon was reported by Gajdusek concerning the rapid abandonment of indigenous treatments for yaws among newly-contacted New Guinea highland tribes [64]. Although most tribes had several remedies for yaws, an endemic and highly prevalent pathogen, none could contend with the dramatic and unambiguous effect of a single penicillin injection. Reproductive disorders, such as pregnancy complications, are largely absent among recorded disease concepts in our study, although historically have been treated by Trio shamans [28,42].

The adoption of non-indigenous disease associations and/or concepts of causality into the present state of Trio ethnomedicine must additionally be fully considered. Although the eldest healers in this study practiced for years prior to sustained contact, it is unlikely that Trio ethnomedicine has remained unaffected from the exposure to Western medical concepts and practices. It is notable, however, that only 2 of 75 recorded disease conditions (*kankë *[cancer], *marareja *[malaria]) recorded by Trio healers in the course of the study comprise obvious adaptation of non-indigenous medical terminology, supporting that the Trio ethnomedical system may remain, at least in its present state, largely intact. Studies of more acculturated indigenous populations in tropical America commonly include the incorporation of clearly biomedical terminology and concepts in informant disease descriptions [65]. In the case of *marareja*, Trio elders recall the presence of a mild, chronic form of malaria (*kananaman*) since their earliest recollections but contrast it to a more severe form, referred with adoptive terminology as *marareja*, which followed initial incursions into their territories. This form, presumably malignant tertian malaria (*P. falciparum*), is considered more refractory to indigenous treatments, and patients generally elect to initially present to the village health outpost for a blood smear and anti-malarial medication.

The wide elective utilization of traditional health services by Trio communities, across all age groups (Figure 2), throughout the several year study interval merits special comment. Health promotion initiatives attempting to advance or integrate traditional medicine among Amerindian communities in other countries have often proved unsuccessful and not widely adopted by communities [66]. The adoption of simple record forms for the surveillance of traditional medicine, such as developed in our study, may have significant utility in health initiatives to guide in the informed integration of traditional health practices. The World Health Organization, in a review of health promotion initiatives attempting to advance traditional medicine, cites failure of programs to acknowledge experience of traditional healers and provide sufficient operating autonomy [66]. The established autonomy of elder traditional healers in the clinics' operation and direction may have been significant to the acceptance and strong utilization within the Trio communities. Clinic utilization was disproportionately higher in Kwamalasamutu across the entire study interval in comparison to Përëre Tëpu, even adjusted for the larger population of Kwamalasamutu. We postulate that this difference may be in part attributable to a greater relative extent of acculturation in the smaller, but more geographically-accessible, village of Përëre Tëpu.

## Conclusion

The results of the present study support the presence of a comprehensive and highly formalized ethnomedical institution within the Trio culture. The rapid and profound loss of transmission of traditional knowledge and skill-sets to younger generations within indigenous communities has been widely demonstrated in ethnobiologic studies [67,68]. The need for preservation of accumulated medicinal plant knowledge, in particular, has been oft espoused as a powerful argument for biocultural conservation [69,70]. Preservation of knowledge of botanical therapeutics alone, however, is insufficient to maintain indigenous medical self-sufficiency. Trio ethnomedicine, as a healing tradition, is a complex art of diagnosis, examination, communication, ritual and treatment, which cannot be "saved" through the collection of herbarium voucher specimens or the documentation of written inventories but rather only transmitted through active practice. Such embedded knowledge and skills are particularly vulnerable to cultural erosion as are experientially-derived and often highly specialized, as among the Trio, to within a few individuals.

A recounting of the history of the dart poison of the Macushi tribe in the neighbouring country of Guyana provides a valuable lesson on the loss of self-sufficiency of indigenous peoples, who, in the process of acculturation, are frequently caught between an undervalued and disintegrating traditional culture and an inaccessible Western system [70,71]. In 1812, naturalist Charles Waterton travelled to the Kanuku mountains of southern Guyana in search of the famed "flying death" of the Macushi tribe, later returning to England with samples of their toxic curare [72]. The elucidation of curare and subsequent adoption of its semi-synthetic derivatives revolutionized the practice of surgery, securing an important place in the operating rooms of the developed world [73].

In 1981, R.J. Lee, UNESCO representative to Guyana, retraced Waterton's original expedition and was able to locate the approximate village sites where Waterton first observed the preparation of the *wourali *poison, a complex admixture of nine different plant species. Lee writes:

"Little of the southern half of Guyana has changed in the last 2,000 years, let alone the 200 since Waterton's birth. Little, that is, except the people. The Amerindian...has been Christianized and westernized, bullied and cheated. His culture was ridiculed by early European missionaries and has slowly been abandoned. In the Macushi territory, between Toka village and Pirara, none of the men know how to make arrow poison...some of the women can make up a "bush tea" to cure fever, but they are no longer a part of the land" [74].

The Macushi, as with the Trio in neighbouring Suriname, remain today in the remote and generally undisturbed rainforest of the Guiana massif. As most Macushi communities have no steady economic base, even shotgun shells are an expensive and limited resource. Not infrequently, in their absence, the men have to rely once again on traditional weapons for hunting, but without the advantage of poisoned tips [70]. The tribal knowledge of the preparation of curare, along with their self-sufficiency in this regard, disappeared years ago. The Trio, as with many other Amerindian peoples that survived the devastation of European conquest and disease, face enormous health, environmental and social challenges in the setting of limited extrinsic resources. The adoption by health and conservation organizations of programs that retain tribal peoples' medical self-sufficiency through promotion of their traditional medicine systems is urgently needed.

## Competing interests

The authors declare that they have no competing interests. This work was supported through grants from Yale University School of Medicine, Pfeiffer Foundation Fellowship, the World Bank Developmental Marketplace Program, Conservation Food & Health Foundation, as well as unrestricted donations from private individuals to the Amazon Conservation Team.

## Authors' contributions

CH designed the research; CH, MU, AU, MP, GE, JJ performed the field research; CH, MU, JJ analyzed data; CH wrote the paper.

## Authors' information

CH is a physician and was affiliated with Amazon Conservation Team as director for the traditional medicine clinic program in Suriname from its initiation in 2000 to 2005. MU is health program coordinator for Amazon Conservation Team Suriname. AU is a Trio shaman from the village of Kwamalasamutu. MP, an ethnobotanist working with the Trio for over 25 years, is president and co-founder of the Amazon Conservation Team. GE is program director of Amazon Conservation Team Suriname. JJ is a policy advisor at the Suriname Ministry of Health.

## Supplementary Material

Additional file 1 **Traditional medicine clinic record form**. Traditional medicine clinic record form (Katamïimë Ëpipakoro, Kwamalasamutu). Click here for file
