# Supplementary material for: Disease concepts and treatment by tribal healers of an Amazonian forest culture
Source: J Ethnobiol Ethnomed. 2009 Oct 12;5:27. doi: 10.1186/1746-4269-5-27 (PMC2774292; doi:10.1186/1746-4269-5-27)
Supplement: Additional file 1 — Traditional medicine clinic record form. Traditional medicine clinic record form (Katamïimë Ëpipakoro, Kwamalasamutu). [file 1746-4269-5-27-S1.PDF]

Ësenëto inumru:
(Patient archief nummer, Patient record number)

KATAMÏIMË ËPIPAKORO ËËSENËTO KAITA
(PATIENTENKAART TRADITIONELE MEDISCH KLINIEK)

Awainato (datum, date):
Pïjai (sjamaan, shaman): …..
Atïjanme ne (klachten, reason for visit):

Ëpi Tëkaramaen (behandeling, treatment):

Ëjantekening (handtekening, signature) X____

Awainato (datum, date):
Pïjai (sjamaan, shaman): …..
Atïjanme ne (klachten, reason for visit):

Ëpi Tëkaramaen (behandeling, treatment):

Ëjantekening (handtekening, signature) X ______________________________________

Awainato (datum, date):
Pïjai (sjamaan, shaman): …..
Atïjanme ne (klachten, reason for visit):

Ëpi Tëkaramaen (behandeling, treatment):

Ëjantekening (handtekening, signature) X _____________________________________
